# Obesity as a Risk Factor in Pediatric Sepsis: A Retrospective Comparative Study Under the Phoenix Definition

**DOI:** 10.3390/jcm14051568

**Published:** 2025-02-26

**Authors:** Koichi Yuki, Sophia Koutsogiannaki

**Affiliations:** 1 Department of Anesthesiology, Critical Care and Pain Medicine, Cardiac Anesthesia Division, Boston Children’s Hospital, Boston, MA 02115, USA; koichi.yuki@childrens.harvard.edu; 2 Department of Anaesthesia and Immunology, Harvard Medical School, Boston, MA 02115, USA; 3 Broad Institute of Harvard and MIT, Cambridge, MA 02142, USA

**Keywords:** pediatric sepsis, Sepsis-2, Phoenix criteria, organ injury, pediatric obesity

## Abstract

**Background:** The relationship between sepsis outcomes and obesity has attracted significant interest in the medical community. However, this association has not been tested under Phoenix criteria, which represent the new pediatric sepsis definition, defining sepsis as life-threatening organ dysfunction in the setting of infection. **Methods:** A single-center, observational, retrospective study of pediatric sepsis patients from January 2014 to December 2019. The PICU was located within a tertiary pediatric center in the United States. Children more than one month old, but less than 18 years old, with a diagnosis of sepsis were included. **Results:** Six hundred and twenty-seven patients with a diagnosis of sepsis based on the Sepsis-2 definition were identified. Within the cohort, 554 patients met the definition of sepsis under the Phoenix criteria. Patients were classified based on the body habitus as underweight, normal, overweight, and obese. Obese patients had significantly higher mortality compared to the normal weight group (*p* = 0.033). More renal dysfunction was also seen in the obesity group (*p* = 0.0007). No difference in the frequency of identified Gram-positive, Gram-negative bacterial, viral, or fungal sepsis was observed between normal-weight and obese patients. **Conclusions:** In our cohort of pediatric sepsis, obesity was significantly associated with a higher degree of organ dysfunction and mortality. However, no difference in the incidence of identified bacterial, fungal, or viral sepsis was observed.

## 1. Introduction

Sepsis is defined as a severe and potentially life-threatening syndrome caused by the body’s overwhelming and dysregulated response to an infection, leading to widespread inflammation, tissue damage, and organ dysfunction [1]. Prompt recognition and treatment are critical to improving outcomes, as sepsis can progress rapidly to septic shock and multiple organ failures. Since there is not a specific treatment for sepsis yet, effective management involves a combination of early diagnosis, timely administration of broad-spectrum antibiotics, fluid resuscitation to stabilize blood pressure, and addressing the underlying infection source [2,3]. Advanced therapies, such as vasopressors for circulatory support, and organ-specific interventions, such as mechanical ventilation or dialysis, may be required in severe cases [4]. Comprehensive care guided by standardized protocols, such as the Surviving Sepsis Campaign guidelines, plays a pivotal role in improving survival rates and minimizing long-term complications [5]. Identifying risk factors such as weakened immune systems, chronic illnesses, advanced age, or the presence of invasive devices is also vital in preventing sepsis and ensuring early detection in high-risk populations [6,7,8,9]. Obesity has been identified as a significant risk factor for many diseases including cardiovascular disease, type 2 diabetes mellitus (DM), hypertension, stroke, dyslipidemia, osteoarthritis, and some cancers [10,11]. However, when it comes to adult sepsis, the “obesity paradox” has been suggested from numerous studies, where higher body mass index (BMI) has been associated with improved sepsis survival [7,8,9,10,12], although this paradox is not linear, as morbid obesity can worsen the outcome [9].

**Figure 1:**
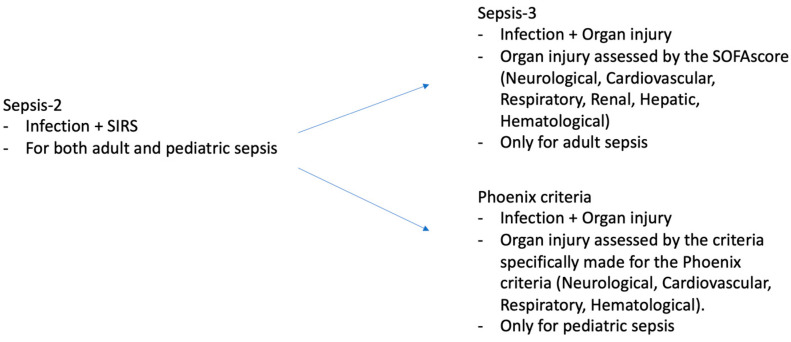
Comparison of Sepsis-2, Sepsis-3, and Phoenix criteria-based sepsis definition. SIRS, systemic inflammatory response syndrome; SOFA, sequential organ failure assessment.

The relationship between obesity and outcomes in pediatric sepsis is complex and less well-defined compared to adult populations [13]. Several factors unique to pediatric sepsis contribute to these inconclusive results, including physiological differences between children and adults, variations in immune responses, comorbidities, developmental physiology, and inconsistencies in study designs [14]. Notably, pediatric studies on obesity and sepsis tend to be smaller in scale and often lack standardization in their definitions of both obesity and sepsis [15,16,17]. A critical distinction between adult and pediatric sepsis lies in their respective definitions, which have evolved differently over time. From 2005 until 2024, pediatric sepsis was defined according to the 2005 International Pediatric Sepsis Consensus Conference. This definition, referred to as Sepsis-2 (Figure 1), was based on systemic inflammatory response syndrome (SIRS) criteria and was shared with adult sepsis at the time [18]. However, the definition of adult sepsis was updated in 2016 to Sepsis-3, which introduced significant changes. Under Sepsis-3, sepsis was defined as a suspected or confirmed infection with life-threatening organ dysfunction, assessed using the Sequential Organ Failure Assessment (SOFA) score, with a threshold of at least two points (Figure 1) [19]. For nearly eight years, these differing definitions created a gap in the criteria for diagnosing sepsis between adults and children. Only recently, in March 2024, a new International Consensus Criteria for Pediatric Sepsis and Septic Shock was introduced, aligning pediatric definitions more closely with Sepsis-3 [20]. This updated definition is based on the Phoenix Sepsis Score, which identifies sepsis in children as a suspected or confirmed infection with life-threatening dysfunction by evaluating four systems—respiratory, cardiovascular, coagulation, and neurological (Figure 1). Septic shock, under this new definition, is characterized as sepsis accompanied by cardiovascular dysfunction indicated by at least one or more points in the cardiovascular organ system. Importantly, as with Sepsis-3, the Phoenix definition requires acute organ dysfunction for the diagnosis of sepsis, making all pediatric sepsis cases equivalent to “severe sepsis” under previous definitions [19,20].

Given the introduction of the Phoenix criteria, it is now possible to re-examine the association between obesity and sepsis in pediatric patients with greater precision. The updated definition, with its emphasis on organ dysfunction, provides a more consistent framework for evaluating outcomes in children. In the current study, we aimed to investigate the relationship between obesity and pediatric sepsis using the new Phoenix criteria. Additionally, we compared these findings to data derived from the previously used Sepsis-2 definition. By doing so, we sought to clarify the role of obesity as a potential risk factor in pediatric sepsis and its outcomes.

## 2. Methods

### 2.1. Study Design

This study was conducted as a retrospective, single-center cohort analysis and received approval from the Institutional Review Board at Boston Children’s Hospital (IRB approval number IRB-P00041981). The requirement for informed consent was waived due to the retrospective nature of the study. The cohort included 806 pediatric patients who were admitted to the intensive care unit (ICU) at Boston Children’s Hospital between January 2014 and December 2019. These patients were identified as having sepsis based on diagnostic codes from the International Classification of Diseases (ICD-9 and ICD-10) and defined according to the Sepsis-2 criteria.

### 2.2. Study Participants and Data Collection

Among the 806 patients enrolled in this study, 627 were younger than 18 years old, but older than one month old. Those less than one month old (newborns) were excluded from our study since they were not included in the initial development and validation of the Phoenix criteria [21]. For these pediatric patients, we collected comprehensive data from the electronic medical record (EMR), including demographic information, vital signs (such as oxygen saturation and mean arterial blood pressure) at the time of ICU admission, and the presence or absence of respiratory support. Additionally, we recorded the length of ICU stay, types of microbes detected (if any), laboratory data (including complete blood count, chemistry panels, liver and kidney function tests, and arterial blood gas analysis), and medications administered during their ICU stay. To evaluate organ dysfunction, we employed the organ dysfunction scoring system used to develop the Phoenix criteria [20]. Initially, eight organ domains were assessed using various established organ injury scoring systems, including the International Pediatric Sepsis Consensus Conference (IPSCC), Pediatric Logistic Organ Dysfunction version 2 (PELOD-2), Pediatric Organ Dysfunction Information Update Mandate (PODIUM), Proulx, and Pediatric Sequential Organ Failure Assessment (pSOFA). These domains included respiratory, cardiovascular, coagulation, neurological, renal, hepatic, immunologic, and endocrine systems. Among these, the immunologic and endocrine systems were assessed exclusively in the PODIUM system. Most organ dysfunction scoring systems, however, consistently evaluated six core domains: respiratory, cardiovascular, coagulation, neurological, renal, and hepatic. Consequently, we focused our analysis on these six domains (Table 1). This was the case for adult sepsis in the Sepsis-3 definition. Neurological status was evaluated based on available documentation. At our institution, the Glasgow Coma Scale (GCS) is not routinely reported unless a patient has an evident neurological issue. Therefore, patients without documented neurological abnormalities were assumed to be neurologically intact. For patients on sedation, neurological status was assessed based on routine pupil reactivity checks. For each domain, we determined the average score during the ICU stay and also identified the maximum score. The Phoenix criteria define sepsis as a Phoenix Sepsis Score of ≥2 points in the presence of suspected infection, with the criteria specifically evaluating the respiratory, cardiovascular, coagulation, and neurological domains [20]. Of the 627 pediatric patients diagnosed with sepsis under the Sepsis-2 definition, 554 also met the Phoenix criteria for sepsis. Pediatric obesity in this cohort was defined based on Body Mass Index (BMI), which estimates weight relative to height. BMI classifications followed the 2000 Centers for Disease Control and Prevention (CDC) growth charts, with underweight defined as a BMI < 5th percentile, normal weight as a BMI between the 5th and 85th percentiles, overweight as a BMI between the 85th and 95th percentiles, and obesity as a BMI ≥ 95th percentile [22].

### 2.3. Statistical Analysis

We presented the number and percentage for categorical variables, mean, and standard deviation (SD) for continuous variables with normal distribution, and median and interquartile range (IQR) for variables with skewed distribution. The Shapiro-Wilk test was used to assess normality. *p* < 0.05 was considered statistically significant. The statistical analysis was performed using STAT13 (College Station, TX, USA).

## 3. Results

### 3.1. Obese Children Had Worse Survival and More Organ Dysfunction Under Sepsis-2 Definition

**Figure 2:**
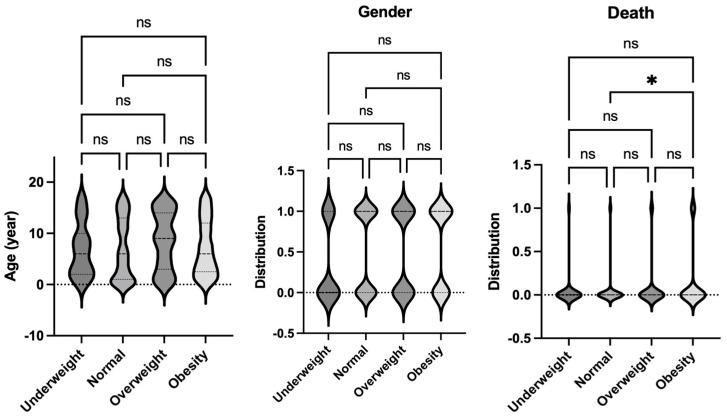
Comparison of demographics and mortality of septic patients based on Sepsis-2 per body habitus. Age, gender, and mortality were compared among underweight, normal, overweight, and obese patients. Dunn’s multiple comparison tests were used for age. For gender and mortality, logistic regression analysis was performed. * *p* < 0.05. ns = not significant.

**Figure 3:**
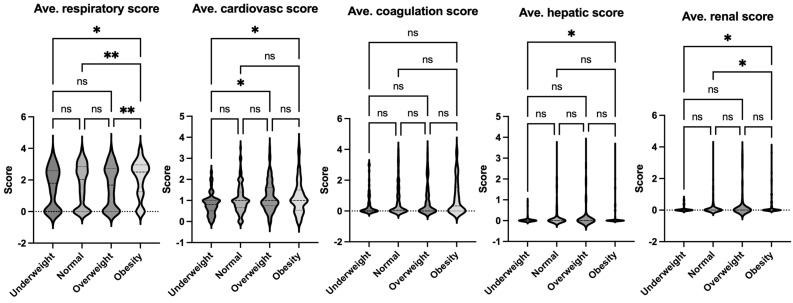
Comparison of average organ scores of septic patients based on Sepsis-2 per body habitus. Dunn’s multiple comparison tests were used. * *p* < 0.05, ** *p* < 0.01. ns = not significant.

**Figure 4:**
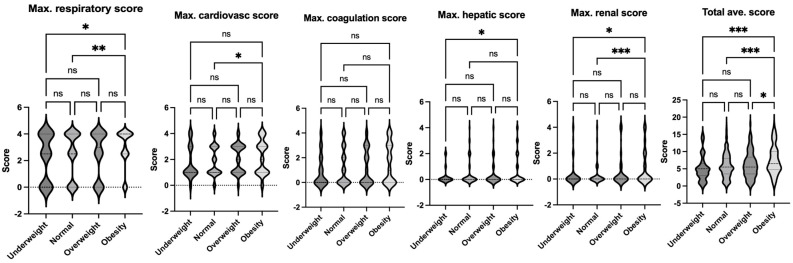
Comparison of maximum organ scores of septic patients based on Sepsis-2 per body habitus. Dunn’s multiple comparison tests were used. *, **, and *** denote *p* < 0.05, <0.01, and <0.001, respectively. Ns = not significant.

The data on pediatric sepsis, defined using the previous Sepsis-2 definition, is summarized in Table 2. The study analyzed patient characteristics and outcomes based on body habitus, categorizing patients into four groups: underweight, normal weight, overweight, and obese. No significant differences in age were observed among these groups. However, the underweight group had a higher proportion of female patients compared to other groups. Notably, the obese group exhibited a higher mortality rate compared to the normal-weight group (Figure 2). This finding aligns with observed differences in organ injury scores. Specifically, the average respiratory and renal organ injury scores during hospital admission were significantly higher in the obese group compared to the normal weight group (Figure 3). These findings suggest that obesity may exacerbate the severity of respiratory and renal dysfunction in pediatric patients with sepsis. Neurological impairment associated with infection was not apparent in this cohort, as no significant differences in neurological scores were identified among the groups. When analyzing the worst organ injury scores recorded during the hospital stay, similar trends persisted (Figure 4). Additionally, the total worst organ injury score and the worst cardiovascular score were significantly worse in the obese group than in the normal weight group (Figure 4), further corroborating the higher mortality observed in obese patients (Figure 2). These findings highlight the association between obesity and worse clinical outcomes, including higher organ injury scores and increased mortality, in pediatric patients with sepsis.

### 3.2. Obese Children Also Had Worse Survival and More Organ Dysfunction Under the Phoenix Criteria

**Figure 5:**
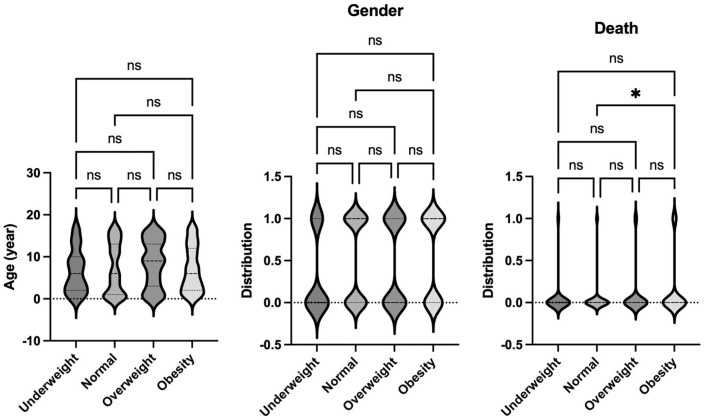
Comparison of demographics and mortality of septic patients based on Phoenix score per body habitus. Age, gender, and mortality were compared among underweight, normal, overweight, and obese patients. Dunn’s multiple comparison tests were used for age. For gender and mortality, logistic regression analysis was performed. * *p* < 0.05. ns = not significant.

We then analyzed the data on pediatric sepsis defined under the newly established Phoenix criteria, which incorporates stricter diagnostic guidelines compared to the previous Sepsis-2 definition. Seventy-three patients who were previously classified as having sepsis under Sepsis-2 did not meet the criteria under the Phoenix system. This refined cohort data is detailed in Table 3. As with the Sepsis-2 analysis, no significant differences in age were identified among the four body habitus groups: underweight, normal weight, overweight, and obese. However, the underweight group again showed a higher proportion of female patients compared to the other groups (Figure 5).

**Figure 6:**
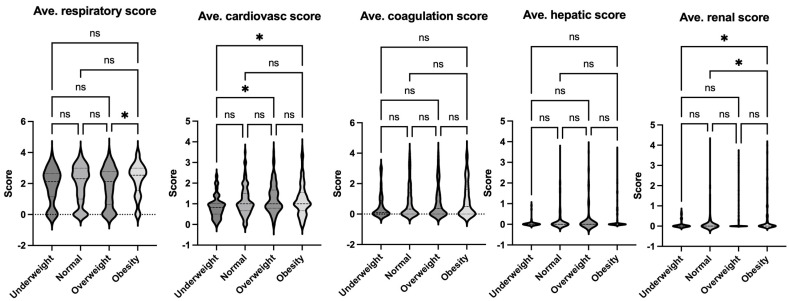
Comparison of average organ scores of septic patients based on Phoenix score per body habitus. Dunn’s multiple comparison tests were used. * *p* < 0.05. ns = not significant.

**Figure 7:**
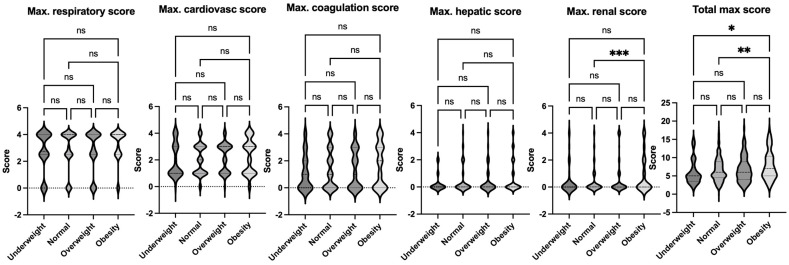
Comparison of maximum organ scores of septic patients based on Phoenix score per body habitus. Dunn’s multiple comparison tests were used. *, **, and *** denote *p* < 0.05, *p* < 0.01, and *p* < 0.001, respectively. ns = not significant.

The obesity group had the highest mortality rate among all groups, consistent with the findings under Sepsis-2 criteria (Figure 5). When examining organ injury scores during admission, the obese group demonstrated significantly greater renal dysfunction compared to the normal weight group, as reflected in the average organ injury scores (Figure 6). This trend persisted when the worst organ injury scores recorded during the hospital stay were analyzed. The obese group consistently exhibited the highest levels of renal dysfunction compared to the normal-weight group (Figure 7). Furthermore, the total worst organ injury score was significantly higher in the obese group than in the other groups (Figure 7), mirroring the higher mortality rate observed in this group (Figure 5). These findings reaffirm that still under the Phoenix criteria, pediatric sepsis patients with obesity exhibit severe clinical outcomes, including increased organ dysfunction and mortality, compared to their non-obese counterparts.

### 3.3. No Difference in the Frequency of Gram-Positive, -Negative, Fungal, or Viral Sepsis Was Identified Based on the Body Habitus

**Figure 8:**
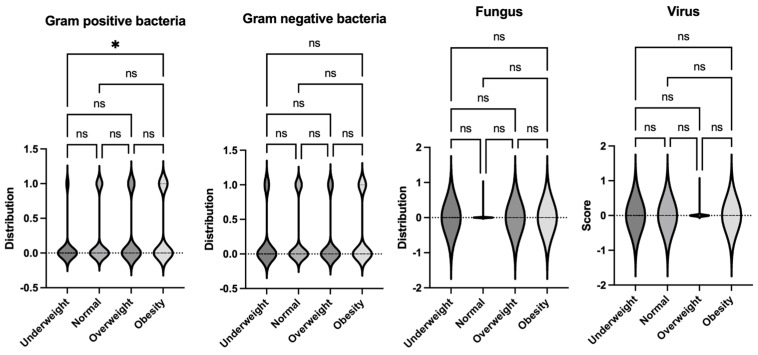
Comparison of frequency of bacterial, viral, and fungal sepsis per Phoenix criteria per body habitus. Logistic regression analysis was performed. * *p* < 0.05. ns = not significant.

Different microbial pathogens interact differently with the host immune system, potentially influencing the severity and outcomes of infections [23]. To assess whether the type of pathogen contributed to differences in clinical outcomes across body habitus groups (underweight, normal weight, overweight, and obese), we analyzed the incidence of these microbes in our cohort. We categorized the identified pathogens based on their type—Gram-positive bacteria, Gram-negative bacteria, fungi, and viruses—and compared their distribution across the body habitus groups (Table 4). While there was a significant number of cases where causative microbes were not identified, no significant differences were observed in the incidence of these pathogens among the groups (Figure 8). The most common Gram-positive bacteria was *Staphylococcus aureus,* followed by *Enterococcus faecalis*, and the most common Gram-negative bacteria was *Pseudomonas aeruginosa*, followed by *Klebsiella pneumoniae*. These findings suggest that body habitus, rather than specific pathogen type, might play a more significant role in influencing morbidity and mortality in pediatric sepsis.

## 4. Discussion

In this study, we identified that the obese pediatric sepsis population had the worst outcome of sepsis compared to the normal-weight group, using, for the first time, the new Phoenix criteria of pediatric sepsis [20].

In line with our results, the study of 454 pediatric ICU (PICU) patients by Peterson et al. reported that although PICU mortality and length of stay were similar for obese/overweight patients (BMI > 85%tile) and normal-weight (5%tile < BMI < 85%tile) critically ill children with sepsis, there was significantly higher use of specialized organ-supportive technology (such as extracorporeal membrane oxygenation (ECMO)) among overweight/obese patients, likely indicating a higher occurrence of multiple organ dysfunction [13]. In accordance, another meta-analysis study showed that critically ill children with obesity exhibited higher mortality compared to patients without obesity. In addition, the length of hospital stay was significantly higher in the group with obesity compared to those without obesity. Duration of ICU stay and need for mechanical ventilation (MV) also tended to be longer in children with obesity, although not statistically significant [15]. It has been stated that even in the lack of significant difference in mortality between groups with and without obesity in severely injured children and adolescents, children with obesity had longer duration of ICU stay and more complications such as sepsis and wound infection [24]. In another study across multiple pediatric intensive care units in the U.S. studying the effect of obesity on mortality in critically ill children, age  > 15 years was associated with increased mortality in children with obesity [25]. Another retrospective cohort from the same group showed no association between obesity status and mortality in children with severe sepsis [26]. However, they found that both overweight and obesity had a higher association in receiving mechanical ventilation and longer intensive care unit stays.

In contrast, one study in the pediatric population using the Kids Inpatient Database (KID) and National Inpatient Sample (NIS) from 2003 to 2014 evaluated the effect of obesity and morbid obesity on outcomes in severe sepsis, suggesting that mortality in the obesity group was lower compared to morbid obesity and control groups [27]. In adult sepsis, the “obesity paradox” has been described, where obesity has been associated with lower mortality in sepsis, with a plethora of clinical studies supporting this idea in intensive care unit patients with severe sepsis and septic shock. An international multicenter cohort study assessing the effect of obesity by BMI on hospital mortality in septic shock patients showed that obese and very obese patients had lower hospital mortality compared to normal-weight patients. However, after adjusting for baseline characteristics and sepsis interventions, the association became non-significant [28]. Another multi-center retrospective study across 139 hospitals in the U.S. showed that BMI was inversely associated with mortality in 55,000 adults with sepsis [29]. In a meta-analysis using four retrospective (n  =  6609 patients) and two prospective (n  =  556) studies, it was also shown that overweight or obese BMIs reduce adjusted mortality in adults admitted to the ICU with sepsis, severe sepsis, or septic shock. A very recent retrospective cohort study also showed that unintentional weight loss was positively associated with a greater risk of mortality in critically ill patients with sepsis in the ICU [30]. One more retrospective study in sepsis revealed patients with a low BMI had increased mortality [31]. In agreement with the sepsis data, the concept of obesity paradox has been reported in various other disease populations, such as acute respiratory distress syndrome, stroke, chronic renal disease, heart failure, chronic obstructive pulmonary disease, cirrhosis, and metastatic malignancy [32,33,34,35,36,37].

Many different hypotheses have been proposed to explain this protective effect of obesity. On the one hand, excess adipose tissue fat stores may serve as a reservoir of energy to be used during the catabolic state created by sepsis-related inflammation [38,39]. Other possible explanations are related to the immunomodulatory effect of the adipose tissue and possible differential sepsis-related systemic inflammatory responses among patients with obesity compared to the control population, leading to dampening of the pro-inflammatory mediators and contributing to a favorable outcome [38,40]. It has also been suggested that there could be a selection bias among patients with obesity, with severe sepsis being cared for in intensive care units, meaning they end up being recipients of more aggressive therapy in anticipation of a difficult clinical course, which may partly explain the obesity paradox [27]. Many experts also state that the “obesity paradox” entity is merely based on most observational studies as a result of selection bias and other methodological errors rather than a true entity, and they discourage the use of this terminology [41,42]. Hence, it is important to acknowledge the limitations, the influence of confounders, and unmeasured entities (genetic factors, body composition, immune response, and illness severity) on outcomes, which might contribute to the findings in our study. On top of that, some more clinical studies have come to challenge the “obesity paradox”. For instance, a retrospective cohort analysis in sepsis suggested that obese and morbidly obese experienced decreased mortality risk vs. normal BMI; however, after adjustment for baseline characteristics, this was no longer significant. There was no significant difference in length of stay (LOS) in the ICU across BMI groups. Neither LOS nor adjusted 28-day mortality was significantly increased or decreased in underweight or obese patients with severe sepsis. It was suggested that morbidly obese patients may have decreased 28-day mortality, partially due to differences in initial presentation and source of infection [43]. Another study investigating the possible impact of obesity, as assessed by the BMI, on morbidity and mortality in ICU patients included in the European observational sepsis occurrence in acutely ill patients (SOAP) study suggested that obesity is associated with increased morbidity but not mortality in critically ill patients [44].

More recently, a meta-analysis study of genome-wide association studies exploring the relationship between life course adiposity and sepsis in both childhood and adulthood suggested that adiposity in childhood and adulthood had causal effects on sepsis incidence. Specifically, using the Mendelian randomization (MR) analysis of inverse variance weighted method revealed that genetic predisposition to increased childhood BMI (OR = 1.29, *p* = 0.003), childhood obesity (OR = 1.07, *p* = 0.034), adult BMI (OR = 1.38, *p* < 0.001), adult waist circumference (OR = 1.01, *p* = 0.028), and adult visceral adiposity (OR = 1.53, *p* < 0.001) predicted a higher risk of sepsis [22].

In addition, the majority of the animal data have associated obesity with more organ injury [45,46], which is in line with our findings in the pediatric population. A recent review on diet-induced obesity in murine models found that among seventeen studies used, with the majority of them using male C57BL/6 mice, cecal ligation, and puncture to induce sepsis, and high-fat diets to induce obesity, seven (64%) studies reported increased mortality in obese septic mice, one (9%) observed a decrease, and three (37%) found no significant difference. The liver, lungs, and kidneys were the most studied organs. Alanine transaminase results were inconclusive. Myeloperoxidase levels were increased in the livers of two studies and inconclusive in the lungs of obese septic mice. Creatinine and neutrophil gelatinase-associated lipocalin were elevated in obese septic mice [47]. Interestingly, obese patients had more respiratory and renal dysfunction. Obese patients tend to require more intrathoracic pressure to maintain the same level of ventilation compared to normal habitus patients. Obese patients have more body mass, which may lead to higher creatinine release.

Lastly, our study showed that the worst sepsis outcome observed in this study associated with obesity could not be attributed to differences in the type of pathogens that caused the infection, in line with a previous study [13], suggesting more intrinsic host mechanisms associated with obesity in sepsis. Overall, the lack of variation in pathogen incidence by body habitus underscores the importance of focusing on host factors, such as body habitus and its physiological implications, and in understanding the mechanisms driving differences in morbidity and mortality in pediatric sepsis. Although our results showed higher mortality under both the Sepsis-2 definition and the Phoenix criteria, the mortality observed under the Phoenix criteria (Table 3) was higher in all the body habitus groups than the one by Sepsis-2 (Table 2), suggesting that the Phoenix criteria would be more suitable to detect the infection process with higher mortality. As the issue around the Sepsis-2 definition was not capturing the disease process leading to higher morbidity and mortality, we can support that the use of the Phoenix criteria would be suitable for the pediatric sepsis definition. Certainly, our study did not address the underlying pathology of worsened outcomes in the obese pediatric cohort, which would provide further and more tailored guidelines on how to treat sepsis in obese pediatric patients, but there is a report that obesity drug glucagon-like peptide 1 receptor (GLP1R) agonists may be associated with lower mortality in sepsis [48], suggesting a potential mechanism. Further research could investigate how these host factors interact with immune responses to infections and whether targeted interventions for specific body habitus groups might improve outcomes.

## 5. Limitations

Single-center studies are valuable for providing detailed insights into specific populations or settings providing controlled conditions and detailed data collection. However, they have some limitations that we need to take into consideration when interpreting the results. Findings from a single-center study may not reflect an easy or straightforward generalization due to differences in patient demographics, local healthcare practices, resources, or infrastructure. The small sample size can limit the statistical power and the ability to detect significant effects or trends.

In addition, the observational and retrospective nature of the study can identify associations but cannot establish cause-and-effect relationships due to the absence of controlled interventions. Uncontrolled confounders, such as unmeasured factors that influence both exposure and outcome, can bias the results as well. In addition, the study’s limitations include using weight and laboratory data only from the first day of PICU admission, preventing analysis of how daily changes affect outcomes and resource use. Accurate height assessment in critically ill neonates and infants (aged 0–2 years) is also challenging, with supine length being the most common method for evaluating stature in this group. Additionally, the lack of data on mortality and morbidity beyond PICU discharge limits the evaluation of weight status on readmissions, late mortality, long-term functional outcomes, and resource use among survivors. Also, this study excluded newborns since they were not included in the initial development and validation of the Phoenix criteria [21]. More studies are needed to evaluate the effect of obesity in newborns with sepsis. 

## 6. Conclusions

In conclusion, this study underscores the critical association between obesity and poor outcomes in pediatric sepsis, particularly when analyzed under the newly introduced Phoenix definition. Our findings reveal that obese children with sepsis experience increased mortality, greater organ dysfunction, and higher organ injury scores compared to their normal-weight and over-weight counterparts, aligning with previous literature on the adverse effects of obesity in critically ill pediatric populations. The study highlights the need for standardized definitions and larger, multi-center investigations to validate these findings and address existing controversies surrounding the “obesity paradox” observed in adult and pediatric sepsis. Additionally, the study’s limitations, regarding its single-center, retrospective design emphasize the necessity for future research to explore the long-term impact of obesity on pediatric sepsis outcomes and identify targeted interventions for this vulnerable population. These insights have the potential to guide more effective management strategies and improve survival rates in obese children with sepsis.

## Figures and Tables

**Table 1:** Respiratory, cardiovascular, coagulation, hepatic, renal, and neurological dysfunction scoring systems originally tested for the development of Phoenix criteria.

Variables	0	1	2	3
Respiratory (0–3 points)				
PaO_2_/FiO_2_	>400	<400 +RS	100~200 + RS	<100 + RS
Or				
SpO_2_/FiO_2_	>292	<292 +RS	148–220 + RS	<148 + RS
Coagulation (0–2 points)		1 point each	max 2 points	
Platelet (×10^3^/µL)	>100	<100		
INR	<1.3	>1.3		
D-Dimer (mg/L)	<2	>2		
Fibrinogen (mg/dL)	>100	<100		
Hepatic (0–1 point)				
Bilirubin (mg/dL)	<4	>4		
ALT (IU/L)	<102	>102		
Cardiovascular (0–6 points).		1 point each	2 points each	
Vasoactive drugs	No	1		
Lactate < mmol/L)	<5	5–10.9	2~	
MAP			>11	
<1 mo	>30	<17–30	<17	
1–11 mo	>38	<25–38	<25	
12–23 mo	>43	<31–43	<31	
24–59 mo	>44	<32–44	<32	
60–143 mo	>48	<36–48	<36	
144–216 mo	>51	<38–51	<38	
Neurologic (0–2 points)				
GCS	>10	<10		
Pupil reactivity	Reactive			Fixed bilaterally
Renal (0–1 point)				
Creatinine (mg/dL)				
<1 mo	<0.8	0.8>		
1–11 mo	<0.3	0.3>		
12–23 mo	<0.4	0.4>		
24–59 mo	<0.6	0.6>		
60–143 mo	<0.7	0.7>		
144–216 mo	<1.0	1.0>		

RS, respiratory support; ALT, alanine aminotransferase; mo, month; MAP, mean arterial pressure; GCS, Glasgow Coma Score.

**Table 2:** Sepsis-2 definition-based data. Data was shown as median (25%tile, 75%tile). m = male, f = female.

	Underweight (n = 51)	Normal (n = 323)	Overweight (n = 112)	Obesity (n = 141)
Age	6 (2, 10)	6 (1, 13)	9 (3, 14)	6 (2.5, 12)
Gender	m 18, f 33	m 168, f 155	m 57, f 55	m 80, f 61
Avg. Respiratory score	1.80 (0, 2.58)	2.03 (0, 2.84)	1.68 (0, 2.73)	2.50 (1.25, 2.96)
Avg. Cardiovasc score	0.80 (0.50, 1.00)	1.00 (0.67, 1.40)	1.00 (0.75, 1.61)	1.00 (0.54, 1.50)
Avg. Coagulation score	0 (0, 0.50)	0.06 (0, 1.04)	0 (0, 1.00)	0.33 (0, 1.55)
Avg. Hepatic score	0 (0, 0)	0 (0, 0)	0 (0, 0)	0 (0, 0.15)
Avg. Renal score	0 (0, 0)	0 (0, 0)	0 (0, 0)	0 (0, 0.28)
Max. Respiratory score	2.5 (0, 4)	2.5 (0, 4)	3 (0, 4)	4 (2.5, 4)
Max. Cardiovasc score	1 (1, 3)	1 (1, 3)	2 (1, 3)	2 (1, 3)
Max. Coagulation score	0 (0, 2)	1 (0, 2)	0 (0, 3)	1 (0, 3)
Max. Hepatic score	0 (0, 0)	0 (0, 0)	0 (0, 0)	0 (0, 1)
Max. Renal score	0 (0, 0)	0 (0, 0)	0 (0, 0)	0 (0, 2)
Total score	5 (3, 6.5)	5.5 (3.5, 8.0)	5.8 (3.5, 8.4)	6.5 (4.8, 10)
Death (%)	2 (3.9%)	16 (5.0%)	8 (7.1%)	18 (12.8%)

**Table 3:** Phoenix criteria-based data. Data were shown as median (25%tile, 75%tile). m = male, f = female.

	Underweight (n = 42)	Normal (n = 280)	Overweight (n = 100)	Obesity (n = 132)
Age	6 (2, 10)	6 (1, 13)	9 (3, 13)	6 (2, 12)
Gender	m 14, f 28	m 145, f 135	m 48, f 52	m 74, f 58
Avg. respiratory score	2.13 (1.15, 2.64)	2.31 (0, 2.99)	2.13 (0.64, 2.77)	2.53 (1.54, 2.99)
Avg. Cardiovasc score	0.82 (0.50, 1.01)	1.00 (0.67, 1.50)	1.00 (0.79, 1.67)	1.00 (0.67, 1.55)
Avg. Coagulation score	0.09 (0, 0.89)	0.33 (0, 1.40)	0.36 (0, 1.25)	0.50 (0, 1.60)
Avg. Hepatic score	0 (0, 0)	0 (0, 0)	0 (0, 0)	0 (0, 0.19)
Avg. Renal score	0 (0, 0)	0 (0, 0)	0 (0, 0.06)	0 (0, 0.38)
Max. Respiratory score	2.8 (2.5, 4)	4 (2.5, 4)	3.5 (2.5, 4)	4 (2.5, 4)
Max. Cardiovasc score	1 (1, 3)	2 (1, 3)	3 (1, 3)	3 (1, 3)
Max. Coagulation score	1 (0, 2)	1 (0, 3)	1 (0, 3)	2 (0, 3)
Max. Hepatic score	0 (0, 0)	0 (0, 0)	0 (0, 0)	0 (0, 1)
Max. Renal score	0 (0, 0)	0 (0, 0)	0 (0, 1)	0 (0, 2)
Total score	5 (3.5, 7.3)	6 (4.5, 8.9)	6 (4, 8.9)	7 (5, 10.4)
Death (%)	2 (4.8%)	16 (5.7%)	8 (8.0%)	18 (13.6%)

**Table 4:** Frequency of bacterial, viral, and fungal sepsis per Phoenix criteria.

	Underweight (n = 42)	Normal (n = 280)	Overweight (n = 100)	Obesity (n = 132)
Gram-positive bacteria	4 (9.5%)	65 (23.2%)	25 (25.0%)	41 (31.1%)
Gram-negative bacteria	8 (19.0%)	70 (25.0%)	20 (20.0%)	37 (28.0%)
Fungus	0 (0%)	1 (0.4%)	0 (0%)	0 (0%)
Virus	0 (0%)	0 (0%)	1 (1.0%)	0 (0%)

## Data Availability

The datasets used and/or analyzed during the current study are available from the corresponding author upon reasonable request.

## Abbreviations

BMI	Body mass index
CDC	Centers for Disease Control and Prevention
DM	Diabetes mellitus
EMR	Electronic medical record
ECMO	Extracorporeal membrane oxygenation
GCS	Glasgow Coma Scale
IRB	Institutional Review Board
ICU	Intensive care unit
ICD	International Classification of Diseases
IPSCC	International Pediatric Sepsis Consensus Conference
IQR	Interquartile range
KID	Kids Inpatient Database
LOS	Length of stay
MV	Mechanical ventilation
MR	Mendelian randomization
NIS	National Inpatient Sample
PICU	Pediatric Intensive Care Unit
PELOD-2	Pediatric Logistic Organ Dysfunction version 2
PODIUM	Pediatric Organ Dysfunction Information Update Mandate
pSOFA	Pediatric Sequential Organ Failure Assessment
SOFA	Sequential Organ Failure Assessment
SOAP	Sepsis Occurrence in Acutely ill Patients
SD	Standard deviation
SIRS	Systemic inflammatory response syndrome
